# Molecular Characterization of *Streptococcus pyogenes* Isolates Recovered from Hospitalized Patients During the Years 2023–2024

**DOI:** 10.3390/microorganisms13092148

**Published:** 2025-09-15

**Authors:** Adile A. Muhtarova, Vasil S. Boyanov, Alexandra S. Alexandrova, Raina T. Gergova

**Affiliations:** Department of Medical Microbiology, Medical Faculty, Medical University of Sofia, Zdrave Str. 2, 1431 Sofia, Bulgaria; v.boyanov@medfac.mu-sofia.bg (V.S.B.); alexandrova_sa@medfac.mu-sofia.bg (A.S.A.)

**Keywords:** *Streptococcus pyogenes*, GAS, *emm* types, *emm* clusters, antimicrobial resistance, inpatients

## Abstract

In recent years, the incidence of severe *Streptococcus pyogenes* (group A Streptococcus, GAS) infections has been increasing worldwide, similar to trends observed prior to the COVID-19 pandemic, alongside a rise in antibiotic resistance. In the present study, we identified the circulating 12 *emm* types and 8 clusters of 70 GAS isolates among inpatients, investigated their association with antibiotic susceptibility, and compared these findings with earlier research conducted in our country. The predominant *emm* types and clusters were *emm*1, *emm*3, and *emm*4, and A-C3, E4, and, A-C5, respectively. *emm*1 was the most common among patients with skin and soft tissue infections or pneumonia, while *emm*3 was detected in patients with peritonsillar abscesses. All isolates demonstrated susceptibility to penicillin and linezolid, whereas the prevalence of resistance to macrolides, lincosamides, and tetracyclines was found to be 14.3%, 14.3%, and 18.6%, respectively. A notable change in the distribution of *emm*-types/clusters has been observed, with emm1/A-C3 now identified as the most prevalent, differing from our previous study conducted in the pre-COVID-19 period. Additionally, we noted a decrease in resistance to macrolides attributed to a lower prevalence of *emm*28 clone. The current research is important for monitoring isolates responsible for severe infections, which is crucial for GAS surveillance.

## 1. Introduction

*Streptococcus pyogenes*, also known as group A *Streptococcus* (GAS), is a microbial species known for its high virulence, leading to infections that often spread on an epidemic scale and result in significant morbidity worldwide [1,2]. The severity of the clinical manifestations of this pathogen exhibits a broad spectrum: ranging from tonsillopharyngitis to purulent skin infections, as well as infections with a systemic course (scarlet fever), and life-threatening (toxic shock syndrome, cellulitis, necrotizing fasciitis, and sepsis) [3,4,5]. Occasionally, streptococcal infections may initially present to be benign; however, an inaccurate diagnosis, improper treatment, or a recurrence of the infection, can result in complications that may lead to disability and a significantly elevated mortality rate. Complications caused by GAS can be divided into two categories: the most common being peritonsillar or other abscesses, which arise from the invasive characteristics of the streptococcal infection. Less frequently, there are non-invasive, non-purulent complications that are challenging to diagnose and treat, specifically post-streptococcal autoimmune complications such as acute post-streptococcal glomerulonephritis and rheumatic fever [6,7,8,9]. In the past twenty years, there has been an increase in both non-suppurative and suppurative complications resulting from GAS infections. This growing impact of diseases can be associated with various factors, such as changes in virulence, the development of antibiotic resistance, and confirmed by the documented increase in GAS infections throughout European countries and other regions [1,5,10,11,12,13].

The ability of GAS to cause disease is associated with various virulence factors, many of which are unique to this species. They can be classified into two categories: cell-wall associated factors and secreted extracellular factors. Each category has distinct effects on tissues, cells, and components of the immune response, as well as impacting biofilm formation [14]. The M protein serves as a crucial virulence factor; therefore, several vaccine candidates are based on it. It is a surface adhesin encoded by the *emm* gene and it is involved in various stages of GAS infections pathogenesis, including adhesion, immune modulation, and tissue invasion [15,16]. According to studies, horizontal gene transfer has been identified as a significant mechanism for generating *emm* gene diversity through recombination, and it contributes to the spread of antibiotic resistance, as certain *emm* variants are associated with specific mobile genetic elements and exhibit high frequency of resistance [1,17,18]. Penicillin continues to be the drug of choice for the empirical treatment of GAS infection, as these bacteria have consistently demonstrated susceptibility to β-lactam antibiotics [19]. Macrolides and lincosamides (ML) are regarded as crucial alternative treatments, particularly for patients who are allergic to β-lactams. Moreover, in cases of severe and extended infections, the standard recommendation is to use lincosamide in combination with penicillin, since clindamycin inhibits the synthesis of GAS exotoxins and the expression of M protein. However, it is important to note that varying levels of resistance to these antibiotics can be observed among different regions [17,19,20].

One of the most commonly used techniques for the epidemiological characterization of GAS is the *emm* typing, based on the sequencing of 180 bp region from the 5′ end of the *emm* gene according to Centers for Disease Control and Prevention (CDC) protocols [21]. At present, the GAS is classified into more than 275 *emm* types, which are grouped into 48 different *emm* clusters [22]. The monitoring of circulating *emm* types and clusters provides substantial epidemiological insights and contributes to understanding the connection between *emm* types and the manifestation of the disease, along with the dissemination of resistant clones. This information will support the future development of a vaccine for GAS [23,24]. The epidemiological investigation of invasive isolates obtained from hospitalized patients suffering from severe infections offers essential insights into population dynamics and strain characteristics linked to emerging lineages, the spread of resistance, and vaccine targets across different geographic regions [8,25,26].

The aim of the study is to identify the circulating *emm* types and clusters of GAS isolates, investigate their association with antibiotic susceptibility, and compare these findings with earlier research conducted on hospitalized patients in our country. This will help in monitoring the evolution of GAS responsible for severe infections, which could be employed in GAS surveillance and future vaccine development.

## 2. Materials and Methods

### 2.1. Data Collection

Between January 2023 to December 2024, a total of 70 GAS samples were collected from two University hospitals located in Sofia (University Hospital ‘Acibadem City Clinic Tokuda’) (72.9%) and Pleven (University Hospital) (27.1%), Bulgaria. The samples were transported and maintained at a temperature of 4 °C. Upon their arrival at the laboratory, microbiological analyzes were conducted systematically. The age range of inpatients was between 3 and 86 years. The majority of the isolates were collected from patients who had been previously diagnosed with infections of the skin and soft tissue (SSTI) (n = 40, 57.1%). These isolates were obtained from purulent wound secretions (62.5%), soft tissue aspirates (25.0%), and erosive skin lesions (12.5%). The distribution of patients’ diagnoses was erysipelas (47.5%), deep soft tissue abscess (25.0%), cellulitis (12.5%), surgical wound infections (10.0%), and necrotising fasciitis (5.0%). The second most common type of sample was obtained from patients who were diagnosed with pneumonia (n = 15, 21.4%). The analysis of pleural fluid (26.7%) and sputum (73.3%) was performed. The remaining isolates were from peritonsillar abscess (n = 12, 17.1%) and perianal abscess (n = 3, 4.3%).

### 2.2. Cultivation and Identification of GAS

All strains were grown in Columbia agar with 5% sheep blood (BD BBL, Becton Dickinson, Franklin Lakes, NJ, USA) at 35 °C in the presence of 5% CO_2_ for 24–48 h. Isolates were confirmed as GAS by routine identification—colony morphology, beta-hemolysis on blood agar plates, Gram staining, positive PYR (Pyrrolidonyl-β-naphthylamide) test, and positive Lancefield group A antigen test (PathoDxtra Strep Grouping Kit, Oxoid™, Thermo Fisher Scientific, Inc., Waltham, MA, USA). GAS isolates were kept frozen at −70 °C in skim milk. *Streptococcus pyogenes* ATCC 21547 was used as a control strain.

### 2.3. DNA Extraction

The isolation DNA was achieved by automated DNA extraction using the MagCore^®^ Genomic DNA Bacterial kit, which is compatible with the MagCore^®^ automated extraction instrument that utilizes magnetic-particle technology (RBC Bioscience Corp., New Taipei City, Taiwan).

### 2.4. Determination of Antibiotic Susceptibility

The Kirby-Bauer disk diffusion method was used to test the susceptibility of all isolates, in accordance with the 2024 EUCAST recommendations (EUCAST, 2024) [27]. This method was performed on Mueller-Hinton agar with 5% defibrinated horse blood and 20 mg/L beta-NAD as antimicrobial agents. The following antimicrobial disks were used: benzilpenicillin G (1 unit), erythromycin (15 μg), clindamycin (2 μg), tetracycline (30 μg), and linezolid (10 μg) (HiMedia, Mumbai, India). The prevalence of macrolide-lincosamide-streptogramin B (MLSB) phenotypes was determined by the double-disk method, whereby erythromycin and clindamycin disks were placed 12–16 mm apart (edge to edge). The plates were then subjected to an incubation process at a temperature of 36 °C for a period of 18 ± 2 h. After incubation, the inhibition zones were measured to determine results based on EUCAST breakpoint rules (EUCAST, 2024) [27].

### 2.5. Molecular Detection of Antimicrobial Resistance Genes

All stains were screened by PCR for the presence of major genes responsible for resistance to macrolides (*mefA, ermA*, and *ermB*) and tetracycline (*tetM* and *tetO*) in GAS. The primer sequences and amplicon sizes have been previously described by Malhotra-Kumar et al. [28]. The following procedure was used to amplify DNA: 30 cycles of initial denaturation at 95 °C for 4 min, annealing at 60 °C for 40 s, extension at 72 °C for 90 s, and final elongation at 72 °C for 7 min.

### 2.6. emm Typing

The entire collection of GAS isolates was analyzed with *emm* typing. The sequencing was performed in accordance with the protocol established by the CDC (https://www.cdc.gov/strep-lab/php/group-a-strep/emm-typing.html, accessed on 10 April 2025). The DNA fragments were subjected to sequencing using the *emm*seq2 primer (5′-TAATCGCTTAGAAAATTAAAAACAGG-3′). Homology was analyzed using a BLAST search, version BLAST+ 2.15.0 (https://blast.ncbi.nlm.nih.gov/Blast.cgi, accessed on 14 April 2025). To extend the research, the data was analyzed using the *emm* cluster typing method. The recognized *emm* types were categorized into related *emm* clusters (https://www.cdc.gov/strep-lab/media/distribution-emm-types.pdf, accessed on 18 April 2025).

### 2.7. Statistical Analysis

Statistical analyses were conducted using IBM SPSS Statistics for Windows v19.0 (IBM Corp., Armonk, NY, USA). Fisher’s exact test was used. A *p*-value ≤ 0.05 was considered statistically significant.

## 3. Results

### 3.1. Molecular Emm Typing

**Figure 1:**
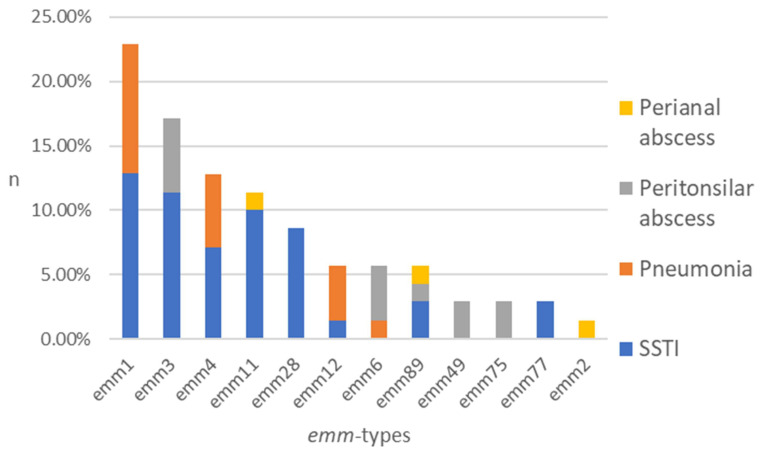
Distribution of *emm* types among the GAS isolates obtained from patient groups represented by stacked columns. n—number of strains in %.

The typing process, including all isolates (n = 70), resulted in the identification of 12 different *emm* types among eight clusters (Figure 1). The five most common types of *emm* were identified as follows: *emm1* (22.9%), *emm3* (17.1%), *emm4* (12.9%), *emm11* (11.4%), and *emm28* (8.6%). The following were present in smaller percentages: *emm12* (5.7%), *emm6* (5.7%), *emm89* (5.7%), *emm49* (2.9%), *emm75* (2.9%), *emm77* (2.9%), and *emm2* (1.4%) (Figure 1).

A total of 40 isolates from SSTI were tested, and the results demonstrated notable heterogeneity, with the following *emm* types identified: *emm*1 (22.5%), *emm*3 (20.0%), *emm*11 (17.5%), *emm*28 (15.0%), *emm*4 (12.5%), *emm*89 (5.0%), *emm*77 (5.0%), and *emm*12 (2.5%). The subsequent *emm* types have been detected in pneumonia samples (n = 15): *emm*1 (46.7%), *emm*4 (26.7%), *emm*12 (20.0%), and *emm*6 (6.7%). A total of 12 isolates from peritonsillar abscesses were examined. The results were as follows: *emm*3 (33.3%), *emm*6 (25.0%), *emm*49 (16.7%), *emm*75 (16.7%), and *emm*89 (8.3%). Three distinct *emm* types were identified from isolates obtained from perianal abscesses (n = 3): *emm*11, *emm*89, and *emm*2 (Figure 1).

### 3.2. Emm-Typing Cluster System

The twelve recognized *emm*-types within the studied population were clearly categorized into eight distinct *emm*-clusters. The distribution of *emm* clusters were: A-C3 (22.9%), E4 (18.6%), A-C5 (17.1%), E6 (14.3%), E1 (12.9%), A-C4 (n = 7, 10.0%), clade_Y_M6 (5.7%) and E3 (2.9%).

Table 1 demonstrates the prevalence of *emm* clusters regarding clinical manifestations, comparing it with our previous study among hospitalized patients conducted between 2014 and 2018 [29]. Regarding the distribution of all strains in both studies, statistical significance was observed for the A-C5 cluster, which was prevalent in 2014–2018 (*p* < 0.05). In the current study, the most common *emm* cluster found in SSTI isolates was E4 (25.0%), followed by A-C3 (22.5%), A-C5 (20.0%), and E6 (17.5%). In samples collected from patients with pneumonia, A-C3 (46.7%) was found to be the most common cluster. In peritonsillar abscess group, A-C5 (33.3%) was the most common, whereas perianal abscess was represented by E4 и E6. Compared to the previous study (2014–2018), statistical significance was found only in the distribution of A-C5 in SSTI group (*p* < 0.05), while there was no significance established for the remaining clinical diagnoses (Table 1). One limitation of comparing the studies is that the earlier study included additional materials from inpatients (n = 34), such as those with meningitis, sepsis, otitis, and TSS (toxic shock syndrome) whereas the current study does not encompass such clinical manifestations.

### 3.3. Antimicrobial Susceptibility Testing

All 70 GAS isolates examined demonstrated susceptibility to both penicillin G and linezolid. The prevalence of resistance to macrolides, lincosamides, and tetracyclines was found to be 14.3%, 14.3%, and 18.6%, respectively. No resistance isolates were identified among the isolates from pneumonia and peritonsillar abscess. In the current study, 90.0% of the strains resistant to macrolides and lincosamides, along with 85.6% of the strains resistant to tetracycline, were isolated from SSTI. There was a statistically significant difference in the distribution of macrolide, lincosamide, and tetracycline resistance among strains isolated from SSTI when compared to other clinical manifestations (*p* < 0.05) (Table 2).

Regarding the genes that determine resistance to ML, we found that *ermB* was present in all resistant strains (n = 10), leading to constitutive MLSB phenotype in each of the isolates. In addition, two isolates were identified to possess both *ermB* and *mefA* gene, while the *ermA* gene was not detected. Concerning the association between *emm*-types/clusters and antibiotic resistance, we observed that the most common among erythromycin-resistant isolates (n = 10) was *emm*28/E4, identified in five strains, followed by *emm*11/E6 in two strains, and *emm*3/A-C5, *emm*1/A-C5, and *emm*12/A-C4 in one strain each. The *mefA* gene was identified in *emm*3 and *emm*12, when all resistant isolates contained *ermB* as mentioned above.

The isolates demonstrating resistance to tetracycline were found to be positive for the *tetM* and *tetO* genes at rates of 76.9% and 23.1%, respectively. The distribution of *emm*-types/clusters among tetracycline-resistant isolates was: *emm*1/A-C3 (4), emm11/E6 (3), *emm*28/E4 (3), *emm*3/A-C5 (2), and *emm*49/E3 (1). The *tetM* gene was identified in all listed *emm*-types, whereas *tetO* gene was present in *emm*1, *emm*11 and *emm*28. Two isolates from *emm*28 and *emm*11 demonstrated resistance to ML and tetracyclines.

## 4. Discussion

Infections caused by GAS can range from mild to severe, potentially resulting in immune-mediated complications, and pose a significant public health challenge, despite the advancements achieved in healthcare. Individuals in hospitals and facilities that host large groups of people encounter an increased risk of developing infections and complications due to factors such as immunocompromising conditions, overcrowding, and elevated levels of social interaction [4,5,30]. The dissemination of highly virulent and resistant strains in these institutions highlights the need to improve surveillance strategies designed to prevent and manage human GAS infections [14]. The most common method for epidemiological surveillance and identifying shifts in the geographical distribution of disease patterns is the *emm*-typing and clustering [31]. Notable variations in the prevalence of *emm* types have been observed in different regions and counties. The worldwide disparities in *emm* types suggest that vaccine targeting particular genotypes may need to be tailored to align with the distribution of local strains [32,33]. A shift in GAS variants or the appearance of new clones may lead to alterations in the incidence and severity of disease within the community [34]. In Europe, between 2000 and 2017 the predominant *emm* types were consistent across all countries, with *emm*1 being the most prevalent, except in Scandinavian countries where *emm*28 and *emm*89 were frequently observed. Similar results were reported in North and South America, where *emm*1 was identified as the most prevalent [31,35]. Before the COVID pandemic in China, Taiwan, and Hong Kong, the most common genotypes were *emm*12, *emm*1, and *emm*4 [36,37]. In the post-COVID period, the predominant *emm*-type in Europe remains *emm*1, followed by *emm*3, *emm*12, *emm*28, and *emm*89 [38,39]. Recent reports indicate that in Asia, as well as North and South America, the distribution has remained similar to the pre-COVID period; however, there appears to be an increase in the frequency of *emm*1 [40,41,42,43]. In comparison to our previous study conducted in 2014–2018, when analyzing the group of inpatients, in which *emm*3/A-C5 was the predominant type, *emm*1/A-C3 has now emerged as the most prevalent [29]. This indicated a notable change in the distribution of *emm*-types/clusters, which is crucial for GAS monitoring in our region. Additionally, the prevalence of *emm*1 is concerning due to its increased virulence and its association with more serious infections [39].

Globally, the prevalence of SSTIs has been reported to be as high as 32.0% of all infections, with GAS being one of the causative agents that result in challenging infections due to its potential for rapid progression and development of complications [44,45]. In the present work, the majority of the isolates (57.1%) were obtained from patients diagnosed with SSTI. In addition to deep soft tissue abscess, cellulitis, surgical wound infections, and necrotising fasciitis, we included 19 strains from patients who were diagnosed with erysipelas. Despite being a superficial infection that affects the outer layers of the skin, numerous studies indicate that erysipelas is a subtype or variant of cellulitis due to its significant involvement and spread through lymphatic vessels, which may result in severe or even life-threatening complications [46,47,48,49,50]. Certain *emm*-types are associated with the expression of various virulence factors, which affect the severity of the diseases they cause. The *emm*1 and *emm*3 GAS strains have been consistently linked to an increased risk of invasive complications, while *emm*4 are generally less correlated with severe disease [51]. The occurrence of rare *emm*-types has been identified as prevalent in SSTI in earlier studies [52,53]. In our research, *emm*1 and *emm*3 together accounted for 42.5%, while in the previous study, they constituted 76.2% [29]. This indicates a reduction in these virulent *emm*-types; however, the rise in less prevalent *emm* types indicates diversity, contributes to understanding epidemic patterns, and is essential for the advancement of vaccines development.

In recent studies, it was proposed that GAS is one of the most common causes of bacterial pneumonia, following *S. pneumoniae* in particular geographic regions, and that leads to more severe disease progression, thereby requiring prolonged hospitalizations [54]. In our study regarding the isolates obtained from inpatients with pneumonia, *emm*1 was found to be the most common, which is consistent with results from other post-COVID-19 research [55,56]. Additionally, our study included 15 isolates obtained from peritonsillar and perianal abscesses. Previous studies have established that GAS is the main causative agent associated with the development of abscesses, with possible significant additional sequelae [57,58]. In both our previous and current studies, *emm*-types showed diversity among strains isolated from abscesses [29]. Isolates from perianal abscesses were in limited number in the present study and showed affiliation to different *emm*-types, which does not confirm their association with a specific *emm*-type. In support of these results, other studies on the same specimens have indicated a heterogeneous structure of *emm*-types, such as *emm*3, *emm*89, and *emm*1, without significant distribution [59,60].

In contrast to other streptococci, GAS has consistently shown universal susceptibility to penicillin and subsequent generations of β-lactam antibiotics, due to its lack of ability to produce β-lactamase enzymes [61,62]. GAS is generally highly susceptible to linezolid, as data indicates a 100% susceptibility rate in numerous clinical isolates from different countries [63,64]. Although it may exhibit bacteriostatic properties against certain bacteria, linezolid is generally bactericidal for GAS strains. Furthermore, it exhibits antitoxin properties as a protein synthesis inhibitor, decreasing the production of toxins and virulence factors, thereby establishing this antibiotic as a viable alternative to other antimicrobial agents, including clindamycin, for managing severe infections [65,66,67]. As expected, all tested isolates in the current study demonstrated susceptibility to both penicillin and linezolid. This supports that penicillin serves as an effective empirical treatment antibiotic for GAS infections. However, patients with penicillin allergies are managed with alternative antibiotics, such as macrolides and lincosamides [68].

There are significant differences in the prevalence of macrolide resistance in GAS worldwide, with rates as low as 5.0% in Europe and as high as 90.0% in China [69]. In the pre-COVID-19 period the incidence of macrolide and lincosamide increased significantly reaching levels of 20.0–40.0% and 19.0%, respectively, across several European countries including Bulgaria as highlighted in our previous report from the years 2013–2016 [8,70]. In recent years, a similar trend of increase was observed in other continents, with Asia exhibiting the highest levels of resistance; however, some reports from Western Europe and South Asia indicated a decrease in resistance rates. The changes in the resistance rate have frequently been associated with the shift in the predominant resistant clones. This correlation is noted between certain *emm* types and macrolide resistance phenotypes and/or genotypes, including a demonstrated connection between *ermB* and *emm*28 [8,69]. In Asia, the rise in ML resistance has been linked to *erm*12 (Taiwan), *emm*12 and *emm*1 (China), as well as *emm*12 and *emm*28. Furthermore, four *emm* clusters (A-C4, E1, E6, E2) have been associated with resistance, along with increased invasive potential. As a result, monitoring particular clones could be beneficial in addressing the spread of antibiotic resistance [69,71,72,73]. For this purpose, in the current research, we investigated the genes responsible for the primary mechanisms of resistance to macrolides and clindamycin—the *erm* genes, whose products induce a modification in the binding site on ribosomes, and the *mef* genes that encode efflux pumps, leading to bacterial resistance specifically against macrolides [9,74]. In a previous study among 102 investigated strains, certain *emm* types were associated with ML resistance—*emm*28, *emm*12, and *emm*4 accounted for 57.0% of the isolates examined. *emm1* and *emm*3 were the most prevalent among *mef* positive isolates, while *emm*77 and *emm*12 were found in *ermA*, and *emm*28 was predominant (88.0%) in *ermB* positive isolates [75]. This indicated that the circulation of *emm*28 contributes to the rise in the ML resistance rate and explained the decrease in resistance with more than 25.0% observed in the current study, where this clone is demonstrated in only six strains.

The prevalence of tetracycline resistance observed in this study was lower compared to both the findings from our earlier research and those reported by other studies in the region [8,75,76]. This observation could be associated with the low resistance of ML, since the resistance genes for both groups of antibiotics in GAS are frequently located on conjugative elements, facilitating the horizontal transfer of these gene determinants together [1,5]. The tetracycline-resistant isolates that were analyzed harbored either the *tet*(*M*) or the *tet*(*O*) gene determinant, and the corresponding *emm*-types were distributed heterogeneously without any particular clonal spread.

Limitation statement: The study included data collected from two university hospitals situated in two cities within a relatively short period due to the COVID-19 pandemic. Some of the collected specimens (perianal abscesses) were in limited numbers, but the results disclose the *emm*-types associated with them and allow comparison with data from other studies. Other limitations are the absence of isolates from blood culture and the fact that we do not specifically investigate the presence of M1 UK genetic variant.

The strengths of the study: Despite these limitations, the application of modern techniques and the evaluation of antibiotic susceptibility in isolates derived from hospitalized patients have enhanced our comprehension of the current situation regarding severe GAS infections, which are relatively rare. Furthermore, we compared the obtained data with previous study to monitor the prevalence of *emm* types and clusters, which is crucial for GAS surveillance.

## 5. Conclusions

The present study investigated the *emm*-types and clusters, as well as antibiotic resistance in hospitalized patients suffering from severe GAS infections, representing the first such analysis conducted in western and northern Bulgaria following the SARS-CoV-2 pandemic. We analyzed the data obtained in comparison with the research conducted in 2014–2018 to highlight the differences in the distribution of *emm*-types and clusters between the two periods, thus directing efforts to develop future vaccines against this pathogen in the region. A notable change in the distribution of *emm*-types/clusters has been observed, as *emm1*/A-C3 has now emerged as the most prevalent among inpatients when compared to our previous study conducted in the pre-COVID-19 period. Additionally, we noted a decrease in resistance to ML attributed to a lower prevalence of the *emm*28 clone, which is typically associated with the *ermB* gene.

## Figures and Tables

**Table 1:** Evolution of *emm* clusters prevalence among the GAS isolates regarding clinical manifestations in strains from 2023 to 2024 compared to strains from 2014 to 2018. Clusters are ordered according to their frequency in the current study.

*emm*-Clusters (Types)	Study Years	All Patients (n = 66 */70)	SSTI (n = 21 */40)	Pneumonia (n = 3 */15)	Peritonsillar Abscess (n = 8 */12)	Perianal Abscess (n = 0 */3)	Other Manifestations *** (n = 34 */0)
A-C3 (*emm*1)	2014–2018 *	17 (25.8%)	6 (28.6%)	1 (33.3%)	1 (12.5%)	0 (0.0%)	9 (26.5%)
	2023–2024	16 (22.9%)	9 (22.5%)	7 (46.7%)	0 (0.0%)	0 (0.0%)	0 (0.0%)
	*p*-value **	0.84	0.76	1			
E4 (*emm*2, 28, 77, 89)	2014–2018 *	13 (19.7%)	1 (4.8%)	0 (0.0%)	2 (25.0%)	0 (0.0%)	10 (29.4%)
	2023–2024	13 (18.6%)	10 (25.0%)	0 (0.0%)	1 (8.3%)	2 (66.7%)	0 (0.0%)
	*p*-value **	1	0.08		0.54		
A-C5 (*emm*3)	2014–2018 *	24 (36.4%)	10 (47.6%)	0	3 (37.5%)	0	11 (32.4%)
	2023–2024	12 (17.1%)	8 (20.0%)	0	4 (33.3%)	0	0
	*p*-value **	**0.01**	**0.04**		1		
E6 (*emm*11, 75)	2014–2018 *	4 (6.1%)	2 (9.5%)	1 (33.3%)	1 (12.5%)	0 (0.0%)	0 (0.0%)
	2023–2024	10 (14.3%)	7 (17.5%)	0 (0.0%)	2 (16.7%)	1 (33.3%)	0 (0.0%)
	*p*-value **	0.16			1		
E1 (*emm*4)	2014–2018 *	3 (4.5%)	2 (9.5%)	0 (0.0%)	0 (0.0%)	0 (0.0%)	1 (2.9%)
	2023–2024	9 (12.9%)	5 (12.5%)	4 (26.7%)	0 (0.0%)	0 (0.0%)	0 (0.0%)
	*p*-value **	0.13	1				
A-C4 (*emm*12)	2014–2018 *	2 (3.0%)	0 (0.0%)	1 (33.3%)	0 (0.0%)	0 (0.0%)	1 (2.9%)
	2023–2024	4 (5.7%)	1 (2.5%)	3 (20.0%)	0 (0.0%)	0 (0.0%)	0 (0.0%)
	*p*-value **	0.68		1			
Clyde_Y_M6 (*emm*6)	2014–2018 *	3 (4.5%)	0 (0.0%)	0 (0.0%)	1 (12.5%)	0 (0.0%)	2 (5.9%)
	2023–2024	4 (5.7%)	0 (0.0%)	1 (6.7%)	3 (25.0%)	0 (0.0%)	0 (0.0%)
	*p*-value **	1			0.62		
E3 (*emm*49)	2014–2018 *	0 (0.0%)	0 (0.0%)	0 (0.0%)	0 (0.0%)	0 (0.0%)	0 (0.0%)
	2023–2024	2 (2.9%)	0 (0.0%)	0 (0.0%)	2	0 (0.0%)	0 (0.0%)
	*p*-value **						

* isolates from 2014 to 2018 [29]. ** a *p*-value <0.05 is considered statistically significant. *** 2014–2018 study included additional materials, which the current research does not encompass.

**Table 2:** Distribution of antibiotic resistance according to the disease entities.

Antibiotics	SSTI (n = 40)	Other Diseases				Total Number (n = 70)	*p*-Value * (SSTI/Other Diseases)
		Pneumonia (n = 15)	Peritonsillar Abscess (n = 12)	Perianal Abscess (n = 3)	Total Other Diseases (n = 30)		
Penicillin	0	0	0	0	0	0	
Linezolid	0	0	0	0	0	0	
Macrolides	9 (22.5%)	0	0	1 (33.3%)	1 (3.3%)	10 (14.3%)	**0.036**
Clindamycin	9 (22.5%)	0	0	1 (33.3%)	1 (3.3%)	10 (14.3%)	**0.036**
Tetracyclines	11 (27.5%)	0	0	2 (66.7%)	2 (6.7%)	13 (18.6%)	**0.032**

* a *p*-value <0.05 is considered statistically significant.

## Data Availability

The original contributions presented in this study are included in the article. Further inquiries can be directed to the corresponding authors.
